# Group involvement and self-rated health among the Japanese elderly: an examination of bonding and bridging social capital

**DOI:** 10.1186/1471-2458-13-1189

**Published:** 2013-12-17

**Authors:** Yoko Kishimoto, Etsuji Suzuki, Toshihide Iwase, Hiroyuki Doi, Soshi Takao

**Affiliations:** 1 Department of Epidemiology, Graduate School of Medicine, Dentistry and Pharmaceutical Sciences, Okayama University, 2-5-1 Shikata-cho, Kita-ku, Okayama 700-8558, Japan; 2 Support Center for Medical Cooperation, Human Resource Placement and Career Promotion of Okayama Prefecture, Graduate School of Medicine, Dentistry and Pharmaceutical Sciences, Okayama University, 2-5-1 Shikata-cho, Kita-ku, Okayama 700-8558, Japan

**Keywords:** Social capital, Bonding, Bridging, Self-rated health, Elderly

## Abstract

**Background:**

To date, only a small amount of research on bonding/bridging social capital has separately examined their effects on health though they have been thought to have differential effects on health outcomes. By using a large population-based sample of elderly Japanese people, we sought to investigate the association between bonding and bridging social capital and self-rated health for men and women separately.

**Methods:**

In August 2010, questionnaires were sent to all residents aged ≥65 years in three municipalities in Okayama prefecture (n = 21232), and 13929 questionnaires were returned (response rate: 65.6%). Social capital was measured from survey responses to questions on participation in six different types of groups: a) the elderly club or sports/hobby/culture circle; b) alumni association; c) political campaign club; d) citizen’s group or environmental preservation activity; e) community association; and f) religious organization. Participant perception of group homogeneity (gender, age, and previous occupation) was used to divide social capital into bonding or bridging. Odds ratios (ORs) and 95% confidence intervals (CIs) for poor self-rated health were calculated.

**Results:**

A total of 11146 subjects (4441 men and 6705 women) were available for the analysis. Among men, bonding and bridging social capital were inversely associated with poor self-rated health (high bonding social capital; OR: 0.55, 95% CI: 0.31–0.99; high bridging social capital; OR: 0.62, 95% CI: 0.48–0.81) after adjusting for age, educational attainment, smoking status, frequency of alcohol consumption, overweight, living arrangements, and type-D personality. The beneficial effect among women was more likely limited to bonding social capital (high bonding social capital; OR: 0.34, 95% CI: 0.12–1.00), and the association between bridging social capital and self-rated health was less clear (high bridging social capital; OR: 0.69, 95% CI: 0.44–1.07).

**Conclusions:**

Bonding/bridging social capital could have differential associations with self-rated health among the Japanese elderly depending on the individual’s sex. Considering the lack of consensus on how to measure bonding and bridging social capital, however, we need to carefully assess the generalizability of our findings. Further research is warranted to identify health-relevant dimensions of social capital in different cultural or economic settings.

## Background

In public health, the concept of social capital has merited attention for more than a decade as a potential approach to health promotion [1]. The concept is popular, and some believe that social capital is a panacea to improve health for everyone [2]. Indeed, one of primary criticisms of social capital research in the public health arena has been that researchers have tended to emphasize the positive effects on health [1]. One approach to address this issue is to distinguish between the effects of bonding and bridging social capital; bonding social capital refers to trusting and cooperative relations with members who are similar in terms of social identity (e.g., social class), whereas bridging social capital refers to connections among individuals who are dissimilar [3]. They have been thought to have differential effects on health outcomes, and strong bonding social capital can sometimes be seen as detrimental to health since it may impose a burden on people’s already stressful lives [4]. To date, however, only a small amount of research on bonding/bridging social capital has separately examined their effects on health and most of these studies used race and ethnicity to evaluate bonding and bridging social capital. For example, in a study from the United States [5], bonding and bridging dimensions of social capital were assessed based on the mix of ethnicity in communities. By using cross-sectional data, this study reported that bonding social capital was beneficial to self-rated health and to reducing stress, whereas bridging social capital did not provide a definitive association with these health outcomes, thereby contradicting the theoretical understanding of bonding/bridging social capital. Although this conceptualization of bonding/bridging social capital makes sense in certain social context (e.g., multiethnic nations/groups), it fails to capture meaningful insight in other social contexts, e.g., in an almost ethnically homogenous nation like Japan. To address this concern, a recent community-based study from Japan measured social capital from responses from participants in several groups, and assessed whether each group represented bonding or bridging social capital, measured by diversity of gender, age, and occupation [6]. They found that bridging social capital was associated with better self-rated health in both sexes, whereas the association was less consistent for bonding social capital. Additionally, a differential pattern was found by sex: women benefited more from bridging social capital than men, whereas men may benefit more from bonding capital than women.

Although most developed countries (including Japan) now face multiple challenges associated with population aging and overall population decline, few studies have examined the effects of social capital on health, specifically among the elderly. It has long been hypothesized that the elderly may be particularly vulnerable to the health-enhancing or health-damaging aspects of residential environments [7]. They may also need to rely on community resources for services, and their daily activities (e.g., food shopping, food consumption, recreation, and social interactions) may often take place in the vicinity of their homes. Hence, both their exposure to community conditions and the degree to which those conditions are relevant to their health may be greater than they are for other age groups [8]. For such a reason, the effect of social capital could be important for the elderly. Given the global trend of population aging, there is an interest in further investigating the association between social capital in a community context and health among the elderly in Japan [9-12], which precedes other countries in experiencing a “super-aging” society [13]. The findings would also be of interest as Japan has built its own particular style of social cohesion owing to its history [14,15]. To date, five studies have examined the association between social capital and health among the elderly in Japan, all of which are from the Aichi Gerontological Evaluation Study (AGES) or the Ohsaki Cohort 2006 Study [16-20]. Although three of these five studies examined the effects of social capital on dental health [16,18,19], these previous studies have implied beneficial effects of social capital on general health among the elderly. For example, a cohort study based on the data of AGES suggested that a smaller friendship network was significantly associated with higher all-cause mortality in both sexes [17].

To our knowledge, only one study from China has examined the association between social capital and health in the elderly by distinguishing bonding and bridging social capital. Norstrand and Xu [21] used cross-sectional data from 1250 subjects aged 65 or older and found that bonding social capital (which was measured as trust on family members, friends, neighbors, etc.) is associated with better physical and emotional health among urban residents. By contrast, they found no clear association between bridging social capital (which was measured as the extent to which people help each other in organizations they have participated in) and health outcomes. It is worthy to note, however, that their findings cannot be necessarily applied to social capital in a community context since they operationalized bonding social capital with a measure of trust that does not uniquely refer to communities and bridging social capital with a measure of reciprocity, both in different social contexts.

Accordingly, we sought to investigate the association between bonding and bridging social capital and self-rated health, using a large population-based sample of elderly Japanese people. As remarked above, it would be of a particular interest to investigate their possible differential effects on health among the elderly in a “super-aging” society like Japan. Following a previous study from Japan [6], we conceptualized the two dimensions of social capital from the perspective of group involvement and examined the association for men and women separately. To address an issue of unmeasured confounding between social capital and health, we adjusted for type-D personality in this study, which consists of negative affectivity (i.e., the tendency to experience negative emotions across time/situations) and social inhibition (i.e., the tendency to inhibit the expression of emotions/behaviors in social interaction to avoid being against others) [22,23].

## Methods

### Participants

Data were obtained from the Okayama Mental Health Survey of Elderly People, a cross-sectional complete community survey conducted in three rural municipalities in Okayama prefecture, located in the western part of Japan [24,25]. In August 2010, the Prefecture Government conducted a postal survey of all residents aged 65 or over in the investigated municipalities. Of the 21232 mailed questionnaires, 201 did not reach their target because of an unknown address, the person was deceased or for other reasons. In total, 13929 were returned (response rate: 65.6%). Subjects with missing values on the questions on social capital, self-rated health, sex or age were excluded. Also excluded were those who had not been resident in the municipalities for more than five years. We employed this procedure to hopefully “wash out” the possible effect of social capital of the communities where they used to live. In total, 2783 were excluded, resulting in 11146 subjects (4441 men and 6705 women) available for the present study.

A thorough explanation of the aim of the survey was provided on the cover of the questionnaire. Residents could freely choose not to participate in this survey without penalty. The return of the questionnaire was therefore considered as informed consent for study participation. The investigators obtained data from the Okayama Prefectural Government after removal of personal identifiers. This epidemiological research study was reviewed and approved by the Ethics Committee of the Okayama University Graduate School of Medicine, Dentistry and Pharmaceutical Sciences.

### Measures

#### Group involvement (bonding and bridging social capital)

In common with a previous study [6], the measure of social capital was derived from the responses in the survey to questions on participation in six different types of groups: a) the elderly club or sports/hobby/culture circle; b) alumni association; c) political campaign club; d) citizen’s group or environmental preservation activity; e) community association; and f) religious organization. Note that some of these group activities are not necessarily community-based. Respondents were asked to assess whether each type of group they attended was either homogeneous or heterogeneous in its social composition, thus differentiating each group into bonding or bridging social capital [3,26]. Specifically, the respondents were asked, “Would you say that the composition of the group that you participate in is diverse or similar with respect to gender, age group, and occupational background?” Respondents who specified their groups as ‘similar’ in background were classified as being involved in bonding social capital, while those who specified their groups as ‘diverse’ were categorized as being involved in bridging social capital.

The number of bonding/bridging social involvements was calculated separately, and then summed to provide the total number of group involvements. To evaluate the dose–response relationship between social capital and self-rated health, the number of groups that the respondents were active in were classified into four levels following a previous study [6]: none, one, two, and three or more, which corresponded to ‘none’, ‘low’, ‘middle’, and ‘high’ social capital.

#### Health outcome

Self-rated health was used as a health outcome variable because of its established validity as a predictor of mortality and morbidity in longitudinal studies [27,28]. Self-rated health was measured by a single item: “How would you rate your overall current state of health: excellent, very good, good, fair, or poor?” From this item, a dichotomous outcome variable was created: 1 = fair/poor; 0 = excellent/very good/good.

#### Covariates

In line with previous studies [5,6,29-31], the following variables were considered as relevant confounders: age, educational attainment, smoking status (never/former, current), frequency of alcohol consumption, overweight, and living arrangement. Educational attainment was categorized as junior high school, high school, and college or further education. Frequency of alcohol consumption was categorized as none, one to three times a month, one to six times a week, and every day. Body mass index was calculated from self-reported height and weight, and 25 or higher (in kg/m^2^) was defined as overweight. Living arrangement was categorized as either living with others or living alone.

In addition to these variables, we adjusted for type-D personality since it could act as a common prior cause of low social capital and poor health. Type-D personality was first developed in relation to chronic heart disease outcome [32]. In this study, we assessed type-D personality by using the Japanese version of 14-item Type D Personality Scale, which is comprised of the two components: negative affectivity and social inhibition [25]. The overall Cronbach’s alpha as an indicator of reliability was 0.8823.

### Statistical analyses

Since we employed individual conceptualization of social capital by using group involvement, we used a single-level analytical strategy throughout the study. Meanwhile, when social capital is conceptualized as a group attribute, multilevel models are relevant in social capital research [33]. Sequential logistic regression analysis was used to examine the relationships between bonding/bridging social capital and self-rated health with subjects in the lowest category (zero group involvement) as the reference group. After examining a crude association (Model 1), age (continuous), educational attainment, smoking status, frequency of alcohol consumption, overweight, and living arrangements were adjusted for in Model 2. In addition, we adjusted for type-D personality in Model 3. Finally, we mutually adjusted for bonding and bridging social capital (Model 4). In addition, logistic regression analyses were used to examine the association between each type of group involvement and self-rated health.

Odds ratios (ORs) and 95% confidence intervals (CIs) for poor self-rated health were calculated, and p values of less than 0.05 (two-sided test) were considered statistically significant. All analyses were performed using STATA/SE 11.1 (StataCorp, College Station, TX, USA).

## Results

Of the 11146 subjects, the prevalence of poor self-rated health was 29.95% for men and 29.44% for women. The demographic characteristics of the subjects are shown in Table 1. Men were more likely to be smokers and consumed alcohol more frequently. The proportions of overweight participants and those with type-D personality were similar between the sexes. The number of bonding/bridging group involvements was inversely associated with poor self-rated health in both sexes (p < 0.01; Table 2). This pattern was consistently observed for total group involvement.

**Table 1:** Demographic characteristics of the subjects responding to a questionnaire, Japan, 2010

**Characteristics**	**Men (N = 4441)**	**Women (N = 6705)**
Mean age (SD)	76.13 (6.93)	77.31 (7.54)
Educational attainment (%)		
Junior high school	2092 (47.11)	2927 (43.65)
High school	1721 (38.75)	2816 (42.00)
Some college or more	453 (10.20)	518 (7.73)
Missing	175 (3.94)	444 (6.62)
Smoking (%)		
Never/former	3487 (78.52)	5988 (89.31)
Current	765 (17.23)	104 (1.55)
Missing	189 (4.26)	613 (9.14)
Alcohol consumption (%)		
None	1470 (33.10)	4817 (71.84)
1-3 times/month	510 (11.48)	749 (11.17)
1-6 times/week	957 (21.55)	566 (8.44)
Everyday	1451 (32.67)	195 (2.91)
Missing	53 (1.19)	378 (5.64)
Body mass index (%)		
<25 kg/m^2^	3534 (79.58)	5202 (77.58)
≥25 kg/m^2^	740 (16.66)	1106 (16.50)
Missing	167 (3.76)	397 (5.92)
Live alone (%)		
Yes	473 (10.65)	1,404 (20.94)
No	3798 (85.52)	4958 (73.94)
Missing	170 (3.83)	343 (5.12)
Type-D personality (%)		
Yes	1853 (41.72)	2674 (39.88)
No	2138 (48.14)	3084 (46.00)
Missing	450 (10.13)	947 (14.12)
Self-rated health (%)		
Good health	3111 (70.05)	4731 (70.56)
Poor health	1330 (29.95)	1974 (29.44)

SD, standard deviation.

**Table 2:** The distribution of the number of group involvement and poor self-rated health stratified by sex

**Men**						**Women**				
**Total**			**Poor self-rated health**			**Total**		**Poor self-rated health**		
**N**		**%**^**a**^	**N**	**%**^**b**^	**P**_**trend**_	**N**	**%**^**a**^	**N**	**%**^**b**^	**P**_**trend**_
The number of total group involvement										
0	878	22.27	495	50.05		2032	30.31	899	44.24	
1	830	18.69	270	32.53		1458	21.74	462	31.69	
2	837	18.85	212	25.33		1293	19.28	295	22.82	
3	709	15.96	152	21.44		978	14.59	171	17.48	
4	568	12.79	98	17.25		575	8.58	99	17.22	
5	338	7.61	68	20.12		269	4.01	36	13.38	
6	170	3.83	35	20.59	<0.001	100	1.49	12	12.00	<0.001
The number of bonding group involvement										
0	3058	68.86	1009	33.00		4681	69.81	1554	33.20	
1	905	20.38	231	25.52		1301	19.40	294	22.60	
2	319	7.18	61	19.12		503	7.50	91	18.09	
3	105	2.36	18	17.14		159	2.37	24	15.09	
4	34	0.77	8	23.53		45	0.67	10	22.22	
5	15	0.34	2	13.33		12	0.18	1	8.33	
6	5	0.11	1	20.00	<0.001	4	0.06	0	0.00	<0.001
The number of bridging group involvement										
0	1319	29.70	582	44.12		2752	41.04	1083	39.35	
1	1022	23.01	303	29.65		1677	25.01	461	27.49	
2	822	18.51	191	23.24		1103	16.45	234	21.21	
3	624	14.05	126	20.19		641	9.56	121	18.88	
4	377	8.49	77	20.42		336	5.01	52	15.48	
5	194	4.37	40	20.62		151	2.25	18	11.92	
6	83	1.87	11	13.25	<0.001	45	0.67	5	11.11	<0.001

^a^We show proportions of each category among the total numbers.

^b^We show proportions of those who reported poor health respondents among each category.

Table 3 shows ORs for poor self-rated health associated with each type of group involvement. Most of the group involvements were (though not significant) associated with lower odds for poor self-rated health, even after adjusting for all covariates (Model 3). Overall, the associations were more pronounced among men; we found a total of five statistically significant associations, four of which were for men. Notably, bonding social capital for participating in an alumni association was significantly associated with lower odds of poor self-rated health for both sexes.

**Table 3:** Odds ratios for poor self-rated health associated with each group involvement, stratified by sex

	**Poor health**	**Model 1**		**Model 2**^**a**^		**Model 3**^**b**^	
	**N (%)**	**OR**	**(95% CI)**	**OR**	**(95% CI)**	**OR**	**(95% CI)**
Men							
The elderly club, sports/hobby/culture circle							
No involvement	854 (36.92)	1.00		1.00		1.00	
Bonding social capital	129 (23.45)	0.69	(0.56-0.85)	0.72	(0.54-0.96)	0.75	(0.56-1.02)
Bridging social capital	300 (21.02)	0.51	(0.44-0.59)	0.61	(0.50-0.74)	0.60	(0.49-0.75)
Alumni association							
No involvement	785 (40.93)	1.00		1.00		1.00	
Bonding social capital	160 (18.89)	0.48	(0.40-0.58)	0.55	(0.43-0.70)	0.57	(0.44-0.75)
Bridging social capital	335 (22.27)	0.55	(0.48-0.64)	0.77	(0.63-0.93)	0.83	(0.67-1.02)
Political campaign club							
No involvement	1071 (31.66)	1.00		1.00		1.00	
Bonding social capital	31 (24.80)	0.77	(0.51-1.16)	0.87	(0.50-1.54)	1.06	(0.58-1.92)
Bridging social capital	187 (23.23)	0.66	(0.55-0.79)	0.80	(0.64-1.02)	0.90	(0.70-1.15)
Citizen’s group, environmental preservation group							
No involvement	1027 (33.11)	1.00		1.00		1.00	
Bonding social capital	29 (17.16)	0.47	(0.31-0.71)	0.53	(0.31-0.89)	0.55	(0.32-0.95)
Bridging social capital	224 (22.36)	0.60	(0.51-0.71)	0.92	(0.74-1.14)	1.00	(0.80-1.26)
Community association							
No involvement	808 (39.90)	1.00		1.00		1.00	
Bonding social capital	70 (23.41)	0.71	(0.54-0.93)	0.65	(0.44-0.96)	0.71	(0.47-1.07)
Bridging social capital	408 (20.43)	0.42	(0.37-0.49)	0.62	(0.51-0.75)	0.69	(0.56-0.84)
Religious organization							
No involvement	1076 (31.32)	1.00		1.00		1.00	
Bonding social capital	36 (33.03)	1.16	(0.77-1.74)	1.61	(0.97-2.68)	1.51	(0.87-2.62)
Bridging social capital	183 (23.49)	0.67	(0.56-0.80)	0.77	(0.60-0.98)	0.79	(0.61-1.02)
Women							
The elderly club, sports/hobby/culture circle							
No involvement	1246 (36.68)	1.00		1.00		1.00	
Bonding social capital	206 (20.02)	0.55	(0.47-0.65)	0.68	(0.46-0.99)	0.73	(0.49-1.09)
Bridging social capital	436 (21.67)	0.57	(0.50-0.64)	0.88	(0.66-1.17)	0.94	(0.69-1.29)
Alumni association							
No involvement	1334 (37.03)	1.00		1.00		1.00	
Bonding social capital	200 (18.50)	0.49	(0.41-0.58)	0.64	(0.45-0.93)	0.63	(0.43-0.93)
Bridging social capital	324 (19.95)	0.51	(0.45-0.59)	0.63	(0.45-0.87)	0.75	(0.53-1.05)
Political campaign club							
No involvement	1739 (30.92)	1.00		1.00		1.00	
Bonding social capital	22 (20.75)	0.62	(0.39-1.00)	1.46	(0.38-5.58)	1.36	(0.35-5.27)
Bridging social capital	101 (17.50)	0.48	(0.38-0.60)	0.59	(0.36-0.97)	0.66	(0.39-1.13)
Citizen’s group, environmental preservation group							
No involvement	1658 (32.10)	1.00		1.00		1.00	
Bonding social capital	30 (17.34)	0.49	(0.33-0.73)	0.13	(0.02-1.01)	0.15	(0.02-1.13)
Bridging social capital	153 (16.91)	0.44	(0.37-0.53)	0.82	(0.56-1.20)	0.92	(0.62-1.37)
Community association							
No involvement	1324 (37.67)	1.00		1.00		1.00	
Bonding social capital	108 (20.30)	0.59	(0.47-0.73)	0.86	(0.53-1.41)	0.81	(0.48-1.37)
Bridging social capital	451 (19.20)	0.43	(0.38-0.49)	0.69	(0.51-0.93)	0.77	(0.56-1.06)
Religious organization							
No involvement	1701 (30.56)	1.00		1.00		1.00	
Bonding social capital	27 (21.26)	0.64	(0.42-0.99)	0.86	(0.31-2.36)	0.72	(0.23-2.22)
Bridging social capital	155 (21.89)	0.64	(0.53-0.78)	0.59	(0.38-0.93)	0.67	(0.42-1.07)

CI, confidence interval; OR, odds ratio.

^a^Adjusted for age, educational attainment, smoking habit, alcohol consumption, weight, and living arrangements.

^b^Adjusted for all of the variables included in Model 2, and type-D personality.

Table 4 shows the gender-stratified associations between different levels of bonding/bridging social capital and poor self-rated health. For men, both bonding and bridging social capital were inversely associated with poor self-rated health in Model 3, with apparently similar magnitudes (high bonding social capital; OR: 0.55, 95% CI: 0.31–0.99, high bridging social capital; OR: 0.62, 95% CI: 0.48–0.81). By contrast, for women, bonding social capital was significantly inversely associated with poor self-rated health while bridging social was not (high bonding social capital; OR: 0.34, 95% CI: 0.12–1.00, high bridging social capital; OR: 0.69, 95% CI: 0.44–1.07). After mutually adjusting for bonding and bridging social capital, no substantial change was observed, and bonding social capital remained significantly inversely associated with poor self-rated health in both sexes (Model 4).

**Table 4:** Odds ratios for poor self-rated health associated with social capital stratified by sex

	**Poor health**	**Model 1**		**Model 2**^**a**^		**Model 3**^**b**^		**Model 4**^**c**^	
	**N (%)**	**OR**	**(95% CI)**	**OR**	**(95% CI)**	**OR**	**(95% CI)**	**OR**	**(95% CI)**
Men									
Social capital									
None	495 (50.05)	1.00		1.00		1.00			
Low	270 (32.53)	0.48	(0.40-0.58)	0.70	(0.52-0.94)	0.72	(0.52-0.98)		
Middle	212 (25.33)	0.34	(0.28-0.41)	0.55	(0.41-0.73)	0.55	(0.40-0.76)		
H0069gh	353 (19.78)	0.25	(0.21-0.29)	0.41	(0.32-0.53)	0.46	(0.35-0.61)		
Bonding social capital									
None	1009 (33.00)	1.00		1.00		1.00			
Low	231 (25.52)	0.70	(0.59-0.82)	0.87	(0.70-1.09)	0.88	(0.69-1.12)	0.89	(0.70-1.14)
Middle	61 (19.12)	0.48	(0.36-0.64)	0.54	(0.37-0.80)	0.55	(0.36-0.83)	0.51	(0.34-0.78)
High	29 (18.24)	0.45	(0.30-0.68)	0.46	(0.26-0.83)	0.55	(0.31-0.99)	0.48	(0.26-0.86)
Bridging social capital									
None	582 (44.12)	1.00		1.00		1.00			
Low	303 (29.65)	0.53	(0.45-0.63)	0.80	(0.62-1.04)	0.82	(0.62-1.07)	0.83	(0.63-1.09)
Middle	191 (23.24)	0.38	(0.32-0.47)	0.58	(0.44-0.77)	0.59	(0.44-0.80)	0.59	(0.43-0.79)
High	254 (19.87)	0.31	(0.26-0.37)	0.55	(0.43-0.71)	0.62	(0.48-0.81)	0.59	(0.45-0.77)
Women									
Social capital									
None	899 (44.24)	1.00		1.00		1.00			
Low	462 (31.69)	0.58	(0.51-0.67)	1.04	(0.69-1.57)	1.19	(0.76-1.86)		
Middle	295 (22.82)	0.37	(0.32-0.44)	0.6	(0.39-0.94)	0.72	(0.45-1.17)		
High	318 (16.55)	0.25	(0.22-0.29)	0.49	(0.33-0.74)	0.62	(0.40-0.97)		
Bonding social capital									
None	1554 (33.20)	1.00		1.00		1.00			
Low	294 (22.60)	0.59	(0.51-0.68)	0.78	(0.55-1.11)	0.78	(0.54-1.13)	0.76	(0.53-1.10)
Middle	91 (18.09)	0.44	(0.35-0.56)	0.68	(0.42-1.10)	0.70	(0.42-1.17)	0.65	(0.39-1.09)
High	35 (15.91)	0.38	(0.26-0.55)	0.39	(0.15-1.01)	0.34	(0.12-1.00)	0.31	(0.10-0.89)
Bridging social capital									
None	1083 (39.35)	1.00		1.00		1.00			
Low	461 (27.49)	0.58	(0.51-0.67)	0.91	(0.63-1.30)	0.96	(0.65-1.41)	0.96	(0.65-1.42)
Middle	234 (21.21)	0.41	(0.35-0.49)	0.68	(0.45-1.03)	0.75	(0.48-1.16)	0.72	(0.46-1.12)
High	196 (16.71)	0.31	(0.26-0.37)	0.54	(0.36-0.82)	0.69	(0.44-1.07)	0.62	(0.40-0.97)

^a^Adjusted for age, living arrangement, educational attainment, smoking habit, alcohol consumption, and overweight.

^b^Adjusted for all of the variables included in Model 2, and type-D personality.

^c^After adjusting for all of the variables included in Model 3, we mutually adjusted for bonding and bridging social capital.

## Discussion

By distinguishing bonding and bridging social capital, the present study examined their differential relationships with self-rated health in the Japanese elderly. The findings suggest that in men, both bonding and bridging social capital are inversely associated with poor self-rated health, even after adjusting for relevant covariates. By contrast, in women, the beneficial effect is more likely limited to bonding social capital, and the association between bridging social capital and self-rated health was less clear when adjusting for covariates. The present study complements previous findings by clarifying the most relevant domain(s) of social capital by distinguishing bonding and bridging social capital.

The same questionnaire on social capital was used by Iwase et al. [6] on a general population (20–80 years old), who found that Japanese women benefited more from bridging social capital and men may benefit more from bonding social capital. Interestingly, this pattern was nearly reversed in the present study, which found that elderly men benefit from both bonding and bridging social capital, whereas elderly women benefit from bonding social capital. It is likely that today’s elderly men in Japan sought and enjoyed stronger associations with their colleagues until retirement in the “close-knit” nature of their companies [34]. While increasing emphasis is placed on cooperation and collaboration inside the workplace [35], they have gained more benefit from bonding social capital from ‘similar’ people in their companies, as implied in the proverb “birds of a feather flock together” [36]. When this strong commitment to the company is lost after retirement, however, they may experience a variety of changes in living arrangements, which leads to changes in physical and mental health [37]. While favorable effects of bonding social capital could remain after retirement, the loss of frequent connection with colleagues could create new challenges for them to establish relationships with community residents, resulting in involvement in new groups with greater diversity in composition respect to gender, age, and previous occupation. This may enable them to construct their identities more easily as their skills and experiences are more likely appreciated by other members in the groups, which could impose beneficial effects on their self-rated health.

The findings about elderly women from the present study may be interpreted from the perspective of flexibility to tolerate and acknowledge different opinions: although they could get along with diverse neighbors in early adulthood, their flexibility may decline with age. Another explanation may be that most women subjects had not been employed and that they have been involved in group activities in the communities over a long time. Even though they initially felt that the group was ‘diverse’, it might become more ‘similar’ over time. The adverse effect of bonding social capital tended to be referred in disadvantaged communities [1]. Our study communities are located in general residential area, which may explain the present findings of bonding social capital. Our findings also suggest that the “threshold” for the beneficial effect of bonding social capital is different in men and women: for women, bonding social capital at the middle level was not significantly inversely associated with poor self-rated health, but it was in men. Although the reason remains unclear, there is a possibility of social desirability among women [38], i.e., elderly women may have tended to over-report the number of group involvement, compared to elderly men.

Notably, unlike a previous Japanese study of general population [6], our findings suggest that bonding social capital is inversely associated with poor self-rated health among the elderly men and women, even after adjusting for bridging social capital. It has been suggested that strong bonding social capital may have a harmful effect on health since it imposes a burden on people’s already stressful lives [4]. Thus, our findings suggest that the “dark side” of bonding social capital may be outweighed by its “bright side” among the elderly. This finding may be partly attributed to a cohort effect; our study subjects were born before the end of World War II, and this generation may highly appreciate the tradition of social commitment, considering it as an opportunity to promote their well-being even if they are required to contribute much more to the groups they belong to.

In the examination of the associations between each type of group involvement and poor self-rated health, bonding social capital for alumni associations was significantly inversely associated with poor self-rated health for both sexes. We thus *a posteriori* hypothesize that the elderly tend to benefit from keeping in touch with old friends. Positive interaction and psychological support from old friends, who share the same circumstances, may be health-promoting, making group members feel a sense of solidarity. Furthermore, these group members may even get the urge to make an active contribution to the groups, which may in turn generate a sense of self-worth in them.

One criticism of social capital that has been repeatedly cited is the possibility that the association between social capital and health is confounded by unmeasured common cause(s), e.g., personality, early childhood environment, and genetic factors. To tackle this problem, type-D personality was controlled for as a covariate. To date, no studies have investigated the associations between social capital and health outcome by adjusting for type-D personality. Thus, the present study is expected to yield robust findings between social capital and health. On a related issue, the use of twin studies is increasingly recognized as an important approach in this field. For example, a recent study from the United States examined the association between social capital and health using data from adult twins [39]. In general, twins are likely to share personality and their early childhood environment, which would provide an opportunity to remove confounding bias due to these factors. By eliminating the effect of these confounders, individual-level cognitive social trust was found to be significantly associated with better self-rated physical health.

There are some limitations to the present study. First, the bonding/bridging instrument did not specify by which factor group members were diverse: gender, age, or occupation (i.e., participants were just asked whichever, in sum). Although diversity in each of these dimensions could theoretically be beneficial to health, diversity in occupational background would be less relevant among the elderly compared with working populations. Rather, considering the finding of a randomized controlled trial of intergenerational interaction [40], diversity in age groups may be the first key component to building social capital among the elderly. Further studies are warranted on the precise nature of group member diversity, preferably for each organization. Furthermore, it may be also beneficial to employ more robust assessment of group involvement, by specifying the frequency of participation for each organization. Second, effects of social capital obviously depend on cultural setting, and we need to consider urban/rural differences. Our study was conducted in rural area, then it is necessary for us to interpret the present results in a careful manner when extrapolating to other setting (e.g., urban areas). Third, there is a possibility of selection bias, since the likelihood of responding to the questionnaire may be influenced by the recipient’s level of social participation as well as the recipient’s health conditions. However, even under this constraint, the ORs are not biased when group involvement and self-rated health are non-interacting (i.e., the effect of group involvement on responding to the questionnaire is independent of the effect of self-rated health on responding to the questionnaire [41]). Fourth, owing to the cross-sectional design of our study, a possibility of reverse causation cannot be ruled out. It may be that an elderly person can participate in more groups if his/her health condition is good. Finally, both exposure and outcome were surveyed by the same, self-evaluated questionnaire, which may have resulted in bias away from the null.

## Conclusions

This is one of the first studies that has examined the health effects of bonding and bridging social capital separately in the elderly. The present findings suggest that both bonding and bridging social capital may be relevant to self-rated health of elderly Japanese men. By contrast, the beneficial effects were less pronounced among elderly women, and they may be limited to bonding social capital. Ideally, studies of this kind may help provide health promotion interventions specifically designed for community-dwelling elderly, which may reduce geographical disparities in health within the nation [42,43]. Considering the lack of consensus on how to measure bonding and bridging social capital, however, we need to carefully assess the generalizability of our findings. Further research is warranted to identify health-relevant dimensions of social capital in different cultural or economic settings, and whether the findings from this study are specific to the elderly of rural area.

## Abbreviations

AGES: Aichi gerontological evaluation study; CI: Confidence interval; OR: Odds ratio; SD: Standard deviation.

## Competing interests

The authors declare that they have no competing interests.

## Authors’ contributions

YK performed the statistical analysis and drafted the manuscript. ES planned the study, supervised the data analysis, and revised the manuscript for important intellectual content. TI and HD helped plan the study and contributed to revising the paper. ST supervised the study and contributed to revising the paper. All authors read and approved the final manuscript.

## Pre-publication history

The pre-publication history for this paper can be accessed here:

http://www.biomedcentral.com/1471-2458/13/1189/prepub
